# Appendicitis among Patients Admitted to the Department of Surgery of a Tertiary Care Centre: A Descriptive Cross-sectional Study

**DOI:** 10.31729/jnma.7980

**Published:** 2023-01-31

**Authors:** Kishor Deo, Prajwala Yogi, Aliska Niroula, Sujata Maharjan

**Affiliations:** 1 Department of Surgery, National Academy of Medical Sciences, Mahaboudha, Kathmandu, Nepal; 2 Kathmandu Medical College and Teaching Hospital, Sinamangal, Kathmandu, Nepal; 3 Bajrabarahi Chapagaun Hospital, Bajrabarahi, Lalitpur, Nepal

**Keywords:** *appendectomy*, *appendicitis*, *prevalence*, *surgery*

## Abstract

**Introduction:**

The prevalence of appendicitis is widespread among both adult and pediatric populations. Despite being so common, its diagnosis remains difficult. Initially, acute appendicitis is managed conservatively. To reduce morbidity and mortality, surgery must be performed promptly. The main objective of the study is to find out the prevalence of appendicitis among patients admitted to the department of surgery of a tertiary care centre.

**Methods:**

A descriptive cross-sectional study was conducted among patients admitted to the Department of Surgery of a tertiary care centre from 1 July 2021 to 1 July 2022. Ethical approval was obtained from the Institutional Review Committee (Reference number: 202/2079/80). Convenience sampling was done. The patient admitted to the Department of Surgery during the study period was included. Point estimate and 95% Confidence Interval were calculated.

**Results:**

Out of 2452 patients, the prevalence of appendicitis was 321 (13.09%) (11.75-14.43, 95% Confidence Interval). The mean age of the patients with appendicitis was 31.57±14.14 years and among them, males were 176 (54.83%).

**Conclusions:**

The prevalence of appendicitis among patients admitted to the department of surgery of a tertiary care centre was lower compared to other studies conducted in similar settings.

## INTRODUCTION

Appendicitis is the inflammation of the vermiform appendix. It typically presents within 24 hours of onset, but can also present as a more chronic condition.^1^ The crude incidence of acute appendicitis was 86 per 100,000 per year.^2^ Delay in diagnosis raises the likelihood of a surgical procedure, as well as morbidity, mortality, and management expenses. Despite being so common, its diagnosis remains difficult.^3^ Early intervention is necessary, for patient management.^4^

Still for most practitioners the timely diagnosis of acute appendicitis is challenging, before the occurrence of complications.^5^ At the initial stage of presentation, acute appendicitis is managed conservatively. However, Appendectomy, via open laparotomy through a limited right lower quadrant incision or via laparoscopy, is the standard treatment for acute appendicitis.^6^

The main objective of the study is to find out the prevalence of appendicitis among patients admitted to the department of surgery of a tertiary care centre.

## METHODS

A descriptive cross-sectional study was conducted among patients admitted to the Department of Surgery of the National Academy of Medical Sciences, Mahaboudha, Kathmandu, Nepal from 1 July 2021 to 1 July 2022. Ethical approval was obtained from the Institutional Review Committee (Reference number: 202/2079/80). Patients admitted to the Department of Surgery during the aforementioned study period were included and those with incomplete hospital record data were excluded from the study. A convenience sampling method was used. The sample size was calculated using the following formula:


$$\begin{array}{ll} \text{n} & ={\text{Z}}^{2}\times \frac{\text{p}\times \text{q}}{{\text{e}}^{\text{2}}}\\ & =1.96^{2}\times \frac{0.215\times 0.785}{0.02^{2}}\\ & =1621 \end{array}$$

Where,

n = required sample size
Z = 1.96 at 95% Confidence Interval (CI)
p = prevalence of appendicitis, 21.5%^8^
q = 1-p
e = margin of error, 2%

Hence, Thus, the calculated minimum required sample size was 1621. After adding 10% for the non-response rate and sample size of 1802 was obtained. However, 2452 patients were taken for the study. Data were entered in a Microsoft Excel Version 2016 and analysed with IBM SPSS Statistics 26.0. Point estimate and 95% CI were calculated.

## RESULTS

**Figure 1:**
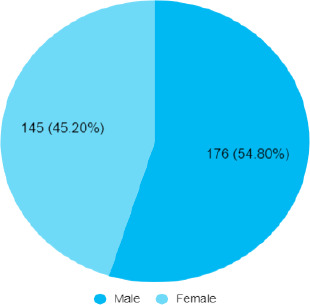
Gender-wise distribution among the patients with appendicitis (n= 321).

Among 2452 patients, the prevalence of appendicitis was 321 (13.09%) (11.75-14.43, 95% CI). The mean age of the patients with appendicitis was 31.57±14.14 years and among them, males were 176 (54.83%) (Figure 1).

Out of the total diagnosed cases, the appendicular lump was present in 26 (8.09%) and appendicular abscess in 7 (2.18%) patients (Table 1).

**Table 1:** Provisional diagnosis of patients with appendicitis (n= 321).

Provisional diagnosis	n (%)
Acute appendicitis	275 (85.93)
Appendicular lump	26 (8.09)
Appendicular abscess	7 (2.18)
Recurrent appendicitis	5 (1.56)
Perforated appendicitis	4 (1.24)
Resolving acute appendicitis	3 (0.93)
Mucocele of appendix	1 (0.31)

Surgical management was patients (Table 2).

**Table 2:** Management of appendicitis (n= 321).

Management	n (%)
Surgical management	161 (50.15)
Conservtive management	160 (49.84)

Among patients who had undergone surgical management, 68 (42.23%) was laparoscopic surgery and among which 1 (1.47%) had undergone interval appendectomy. A total of 93 (57.76%) were open appendectomy surgery. Out of total appendicitis cases, 2 (0.62%) had urinary tract infections (UTIs) (Table 3).

**Table 3:** Co-existing conditions (n= 321).

Conditions	n (%)
Pneumoperitoneum	1 (0.31)
HIV positive	2 (0.62)
Hemophilia	1 (0.31)
Brucellosis	1 (0.31)
UTIs	2 (0.62)
Diabetes mellitus	1 (0.31)

## DISCUSSION

Appendix is characterised as narrow and long, passing upward behind the cecum, to the left behind the ileum and mesentery, or downward and inward into the pelvis.^7^ Inflammation of vermiform appendix leads to appendicitis. Patients can present acutely, within 24 hours of onset, but can also present as a more chronic condition.^1^ One of the commonest causes of acute abdomen presenting to the emergency department is acute appendicitis which requires emergency surgery. The incidence of acute appendicitis was 86 per 100,000 per year.^2^ In our study the prevalence of appendicitis was found to be 321 (13.09%), which is lower than the prevalence of appendicitis in other studies i.e. 21.50%.^8^

In our study, the mean age group was 31.57±14.14 years, whereas in another similar study the mean age was 30.94±15.75 years which is almost similar to our finding.^9^ The most common age for appendicitis was found to be 21-30 i.e. (34.54%) followed by 1120 (26.36%).^8^ Out of 175, 92 (52.6%) were female and the remaining 83 (47.4%) were male in a study done in Nepal.^9^ This study shows that appendicitis is more prevalent in males. Whereas in another study, appendicitis was more prevalent in males, 42 (60%) in males and 28 (40%) in females.^10^ In our study appendicitis is more prevalent in males i.e. the males 176 (54.83%) and females 145 (45.17%).

Delay in diagnosis increases the likelihood of a destructive surgical approach, as well as morbidity, mortality, and management expenses. Despite being so widespread, its diagnosis is still challenging.^3^ Different scoring systems like The Alvarado score, Pediatric Appendicitis Score, MANTRELS score and Appendicitis Inflammatory Response score incorporate common clinical and laboratory findings to stratify patients as low, moderate, or high risk and can help in making a timely diagnosis. Thus, patients can be managed early with proper intervention.^4^ to avoid complications and reduce significant morbidity and mortality, a timely diagnosis of appendicitis is needed and is challenging for practitioners.^5^

Patients mostly present with abdominal pain, in the emergency department. Appendicitis is the most common cause of abdominal pain. The most common feature of appendicitis was pain followed by fever and vomiting. The initial presentation involves periumbilical colicky pain around the midgut. Localised pain coincides with parietal peritoneum irritation. The pain intensifies over a period of 24 hours, accompanied by nausea, vomiting, and loss of appetite.^10^

Other features of acute appendicitis are: right lower quadrant pain, rigidity, migration/ periumbilical pain, pain before vomiting, Psoas sign, fever, guarding, no similar previous pain, rebound tenderness, anorexia, vomiting, rectal tenderness, nausea, Obturator sign, Rovsing Sign, Absent/ decreased bowel sounds, pain with hopping/coughing/percussion.^4^ Ultrasonography, computed tomography (CT), and magnetic resonance imaging are preferred modalities for the evaluation of patients with suspected acute appendicitis.^4^

Initially, acute appendicitis is managed conservatively. In a similar study of Gandaki Medical College Pokhara, out of 1021, 203 (19.88%) were managed conservatively whereas 818 (80.11%) were surgically managed.^11^ In our study, 160 (49.84) were managed surgically while 160 (49.84) were managed conservatively. However, Appendectomy, via open laparotomy through a limited right lower quadrant incision or via laparoscopy, is the standard treatment for acute appendicitis.^6^ Out of 175 cases, 119 (68%) had appendectomy with open 108 (90.75%), and laparoscopic 11 (9.2%). The remaining 56 (32%) were treated conservatively in a study done in Nepal.^9^ In our study, open appendectomy was done in 93 (57.76%) whereas laparoscopic appendectomy was done in 68 (42.23%). This shows that still, open appendectomy is more frequently performed in our settings.

Perforation is the most concerning complication of acute appendicitis and may lead to abscesses, peritonitis, bowel obstruction, fertility issues, and sepsis.^12^ In our study the perforated appendix was present in 4 (1.24%). Acute appendicitis is a common emergency situation so, timely diagnosis of acute appendicitis is the challenging phase for the practitioner to avoid the complication. But still, the data available about aetiology, and intervention are not still clear.

Since the study is a single institution-based descriptive cross-sectional study, the results might not be completely generalizable in other settings. So, a study design with a higher level of evidence is recommended for future studies. There may also be information bias and respondent bias.

## CONCLUSIONS

The prevalence of appendicitis among the patients admitted to the surgery department of a tertiary care centre was lower than in other studies conducted in similar settings.
